# Ischemic stroke as the initial manifestation of atrial fibrillation; is it higher than expected?

**DOI:** 10.34172/jcvtr.2021.35

**Published:** 2021-08-25

**Authors:** Elyar Sadeghi Hokmabadi, Samad Shams Vahdati, Elyar Alizadeh Najmi

**Affiliations:** ^1^Neurosciences Research Center, Department of Neurology, Imam Reza Hospital, Tabriz University of Medical Sciences, Tabriz, Iran; ^2^Emergency Medicine Research Team, Imam Reza Hospital, Tabriz University of Medical Sciences, Tabriz, Iran

Atrial fibrillation (AF) may be asymptomatic and paroxysmal, and thus stroke can be the initial manifestation of AF. AF increases the risk of ischemic stroke by 2.5 times and recurrent strokes in patients with AF are a great burden on the national health care system. On the other hand, patients with asymptomatic AF often refuse treatment and as a result are at increased risk of thromboembolic events, especially ischemic stroke.^1^ The Framingham Heart Study, a comprehensive large cohort community-based study of 1 809 patients with AF heart rhythm, estimated that 2 to 5 ischemic strokes per 10 000 person-years were the first sign of AF heart rhythm.^2^

To assess the burden of ischemic stroke as an initial manifestation of AF, we did a prospective study. In a ten-month period, all patients admitted with acute ischemic stroke in Imam Reza hospital, a tertiary referral educational center in Tabriz, Iran, were enrolled in this study.

During the study period, 810 patients were admitted with acute ischemic stroke, of whom 177 (21.8%) patients had AF heart rhythm. The median age of this group was 76 (IQR: 71-83), and 52% (87) were male. Hypertension was more common in patients in the AF group than those in the non-AF group [125 (70.6%) vs. 393 (62.1%), *P* = 0.036] but the frequency of diabetes mellitus was not significantly different between the two study groups [40 (22.6%) vs. 147 (23.22%), *P* = 0.861].

Face-to-face interviews were done for all patient with AF. Patients with AF heart rhythm were divided into two groups according to the time of the AF diagnosis: 1) previous AF, patients with a known history of AF before the stroke, and 2) new AF, patients with a diagnosis of AF made during the index hospitalization. Of these 177 patients, 44 (24.9%) had a previous history of AF (“previous AF” group) and 133 (75.1%) were diagnosed with AF during the current hospital admission (“new AF” group).

A retrospective large cohort study conducted by Jaakkola et al^3^ in Finland, found that in a group of 3 623 patients with acute ischemic stroke for the first time and AF, 79.2% of patient had “previous AF” and 20.8% had “new AF” heart rhythms1; in our study, about 80% of patients had a “new AF” heart rhythm. This clear difference may be due to two reasons: there is a weak health referral system, especially in rural areas, in our region; and since the visit for a private physician (cardiologist or neurologist) is cheap, most people are not under regular risk factor surveillance within the health system and they prefer to visit physicians directly. This is important, because screening for other risk factors like hypertension or diabetes mellitus is in the first referral level of our health system, where there is a greater chance to recognize asymptomatic AF. Furthermore, due to very crowded private physician offices, there is not enough time for patient education and this results in another problem: some patients with a diagnosis of AF undergo a course of treatment with anticoagulants, but due to the lack of knowledge about the importance of their disease and the need for treatment over time, these patients do not visit their physician again and anticoagulation treatment then stops.

Based on the results of this study, contrary to current beliefs in our region, it seems that the burden of unrecognized AF is much higher than under-treatment of these patients. To decrease this burden, several strategies are recommended, including population-wide AF-screening efforts, pulse check education for patients and their families,^3^ spending more time on education in physician offices providing complete and detailed explanations about AF and its risks, informing patients about medication, side effects of taking and not taking it, and importantly, continuous follow-up of the patients.^4-6^ At the bottom line, the burden of ischemic stroke as the initial manifestation of AF seems to be higher in our region; thus, physicians should be alerted and appropriate interventions should be applied.

## Competing interests

The authors declare that they have no conflict of interests regarding this publication.

## Ethical approval

This study was approved by regional ethic committee of Tabriz University of medical sciences with no.: IR.TBZMED.REC.1397.083

## Funding

This research was supported by Neuroscience Research Center (NSRC), Tabriz University of Medical Sciences.
